# Comparative Analysis of SYNTAX and BCIS Jeopardy Score of Diabetics Versus Non-Diabetic Patients with Complex Coronary Artery Disease

**DOI:** 10.3390/jcm14103433

**Published:** 2025-05-14

**Authors:** Tarek Abdeldayem, Saif Memon, Muntaser Omari, Mohamed Farag, Ayman Al-Atta, Abdalazeem Ibrahem, Tarik Salim, Bilal Bawamia, Mohaned Egred, Mohammad Alkhalil

**Affiliations:** 1 Cardiothoracic Centre, Freeman Hospital, Newcastle-upon-Tyne NE1 7RU, UK; tarek.abdeldayem1@nhs.net (T.A.); saif.memon@nhs.net (S.M.); muntaser.omari@nhs.net (M.O.); mohamedfarag@nhs.net (M.F.); a.al-atta@nhs.net (A.A.-A.); abdalazeem.ibrahem2@nhs.net (A.I.); tariik.salim@nhs.net (T.S.); bilal-reshad.bawamia@nhs.net (B.B.); m.egred@nhs.net (M.E.); 2 Translational and Clinical Research Institute, Newcastle University, Newcastle-upon-Tyne NE1 7RU, UK

**Keywords:** diabetes, coronary artery disease, SYNTAX, BCIS jeopardy score, mortality

## Abstract

**Background:** Diabetic patients tend to have complex coronary artery disease (CAD). Understanding their procedural risk may help to guide treatment strategies. SYNTAX and the British Cardiovascular Intervention Society Jeopardy Score (BCIS-JS) have been used to define complex CAD, but their use has not been compared in diabetic patients. **Methods:** This is a retrospective analysis of prospectively collected data of consecutive patients who underwent complex percutaneous coronary intervention (PCI) and were deemed unsuitable for surgical revascularization. Both SYNTAX and BCIS-JS were calculated by experienced operators who were blinded to patient’s outcome. The primary endpoint was all-cause mortality at 12 months. **Results:** Of 452 patients included in the study, diabetes was present in 35% patients. There was a modest relationship between BCIS-JS and SYNTAX score (Spearman r = 0.44, *p* < 0.001) and this relationship was even weaker in patients with diabetes (Spearman r = 0.32, *p* < 0.001). The primary endpoint was comparable in the non-diabetic group, irrespective of the score system (SYNTAX or BCIS-JS) used to define complex CAD. In contrast, there was a differential prognostic outcome in the diabetic group, whereby the primary endpoint was more frequently reported in diabetic patients with high versus low SYNTAX scores [HR 4.96, 95% CI (1.44–17.03), *p* = 0.011] but not when BCIS-JS was used. **Conclusions:** There was a modest relationship between BCIS-JS and SYNTAX score. Unlike BCIS-JS, the SYNTAX score identified those who are at increased risk of death among diabetic patients. Both scoring systems did not effectively differentiate mortality risk in non-diabetic patients. Future research is needed to confirm this study’s findings.

## 1. Introduction

Coronary artery disease (CAD) remains one of the leading cause for morbidity and mortality globally [1]. Different strategies have been utilised to risk-stratify patients and to aid decision making regarding the optimal mode of revascularization for those who are symptomatic with angina [2,3,4]. The Synergy between Percutaneous Coronary Intervention with Taxus and Cardiac Surgery (SYNTAX) score is a well-established method to grade the complexity of CAD, and a high SYNTAX score is associated with worse clinical outcomes in patients undergoing percutaneous coronary intervention (PCI) at long-term follow-up [5].

The BCIS (British Cardiovascular Intervention Society) Jeopardy Score (BCIS-JS) was proposed as a scoring system, taking into account the at risk myocardium, and is modified from the Duke Jeopardy Score [4,6]. The BCIS-JS is a simple system that provides a semi-quantitative assessment of the myocardium at jeopardy and can be used in patients with previous coronary artery bypass graft (CABG) [7,8]. It may provide insight into the procedural risk of patients undergoing PCI, particularly in patients with impaired left ventricle function [7,8].

Increasingly, BCIS-JS is being utilised as a method of defining complex CAD. Direct comparison between the two scoring systems is limited, particularly in patients who are deemed to have a high revascularization risk, such as those with diabetes [9]. This is important given the unique challenges faced by patients with diabetes and CAD, which include its diffuse nature, the likelihood of having multi-vessel disease, the degree of calcifications, and concomitant co-morbidities such as renal impairment, stroke, and peripheral vascular disease that inevitably contribute to worse clinical outcomes. Moreover, previous studies highlighted the limitations of using existing scoring systems to guide mode of revascularization in patients with diabetes. The data suggested suboptimal clinical outcomes in patients undergoing PCI, irrespective of the calculated SYNTAX score [10].

Nonetheless, recent data suggest a role for the use of functional assessment [11,12], intra-vascular imaging [13], and intensive pharmacotherapy in diabetic patients undergoing PCI [14,15]. A better understanding of procedural and long-term risks, using BCIS-JS and/or SYNTAX scoring systems, will help to provide insights into the role of PCI in patients with diabetes. Therefore, the aim of this study was to assess the relationship between SYNTAX and BCIS-JS in patients who underwent complex PCI procedures and to identify if either of the two scoring systems can provide prognostic insight for patients with diabetes.

## 2. Methods

### 2.1. Study Design

This is a single-centre, retrospective, observational study of consecutive patients who underwent a complex PCI procedure and deemed not suitable for surgical revascularization (formal surgical approach turned down) [16]. Clinical and procedural characteristics were prospectively entered into institutional electronic database and validated by a dedicated data manager. Anonymized data were obtained and retrospectively analysed and therefore the need for informed consent was waived by the institutional Medical Ethical Committee. The study was conducted in accordance with the Declaration of Helsinki and Good Clinical Practice guidelines.

The included patients had discussions and were turned down for surgery after being considered for surgical revascularization by the heart team. The heart team included cardiac surgeons, interventional cardiologists, and general cardiologists. The decision for this treatment method being turned down for them was based on a holistic approach to every individual patient, including a review of their clinical history and presentation, comorbidities, coronary anatomy, and degree of left ventricle function. Importantly, the ability to achieve full revascularization using a percutaneous approach was also factored in before reaching the final consensus. Patients were only included in this analysis if they underwent PCI and those who were treated medically or had plain balloon angioplasty were excluded.

### 2.2. PCI Procedure

The PCI procedure was performed according to current guidelines on coronary revascularization [17,18]. The PCI strategy was left to the operator’s discretion, but the majority of cases were performed using a trans-radial approach, minimal sedation, and local anaesthesia. Heparin was universally used in all cases and patients were pre-loaded with aspirin and a second antiplatelet agent before PCI.

The cohort included both stable and unstable patients. Acute coronary syndrome (ACS) included unstable angina, non-ST elevation myocardial infarction (NSTEMI), and ST elevation myocardial infarction (STEMI). Unstable angina was defined as myocardial ischemia at rest or on minimal exertion in the absence of acute cardiomyocyte injury, identified using cardiac biomarker, that resulted in urgent hospital admission. Myocardial infarction was defined according to the fourth universal definition of myocardial infarction [19].

### 2.3. Study Endpoints

The primary endpoint of the study was all-cause mortality at 12 months follow-up. This was obtained from the institutional local database and cross-checked using data from the Office of National Statistics (ONS). Procedural complication was defined as the composite endpoint of side branch occlusion, slow flow or no-reflow, any arterial dissection, pericardial tamponade, cardiogenic shock, or major bleeding.

Both SYNTAX and BCIS-JS scores were calculated by experienced operators who were blinded to each other scores. The scoring calculation was performed off-line and was performed blind to the patients’ clinical outcomes. In-hospital outcomes and complications were gathered from the databases. A high SYNATX score was defined as more than 33, in line with previously reported studies [3,10,20]. The Revascularization for Ischemic Ventricular Dysfunction (REVIVED) study defined a high BCIS-JS as more than 6 and the current analysis utilised the same cut-off [8].

Medical treatment was assessed at the last clinical appointment or at hospital discharge if patient did not survive or clinical follow-up was not available. Optimal medical treatment was defined as the patient receiving beta blockers and angiotensin-converting enzyme inhibitor (ACEI) or angiotensin II receptor blocker (ARB) for patients with preserved left ventricle (LV) function. For patients with severe LV function, the use of mineralocorticoid receptor antagonist (MRA) was factored in when defining optimal medical treatment.

### 2.4. Statistical Analysis

Data were tested for normality using the Shapiro–Wilk test and reported as mean (+/− standard deviation) for normal distribution variables and compared using unpaired t tests. Non-normally distributed data were reported using median (Q1–Q3) and compared using Wilcoxon’s rank-sum test. Dichotomous data were reported as absolute numbers and percentages, and compared using the χ^2^ test or Fisher’s exact test, as appropriate. Correlation analysis was performed using the Spearman test, given the semi-quantitative nature of BCIS-JS data. The Kaplan–Meier survival methods with log-rank tests were used to assess the role of high versus low SYNTAX and BCIS-JS on one-year mortality. All statistical analyses were performed using SPSS 28.0 and *p* < 0.05 was considered significant.

## 3. Results

A total of 452 patients were included in the study. The mean age was 72 ± 11 years, with 29% of the participants being female. The indication for PCI was 46% for stable angina presentation. Preserved left ventricle function was present in 52% of patients. The mean Euro Score II was 6 (±6) and a large percentage of patients had left main intervention (42%). A high SYNTAX score was calculated in 57% of patients compared to 93% of patients who were deemed to have high BCIS-JS.

The proportion of diabetic patients was 35%. There were no significant differences between diabetic and non-diabetic patients in clinical presentation, co-morbidities, or gender, although non-diabetic patients were older (73 ± 11 vs. 70 ± 11, *p* = 0.001), with lower body mass index (27 ± 5 vs. 31 ± 7, *p* < 0.001). The percentages of previous stroke (13% vs. 6%) or chronic obstructive pulmonary disease (20% vs. 11%, *p* = 0.043) were also higher in patients that were non-diabetic compared to those that were diabetic. Procedural complexity was comparable between diabetic and non-diabetic patient in relation to left main intervention, the number of attempted lesions, number of stents, SYNTAX score, and BCIS-JS score. Baseline clinical and procedural characteristics are presented in Table 1.

**Figure 1:**
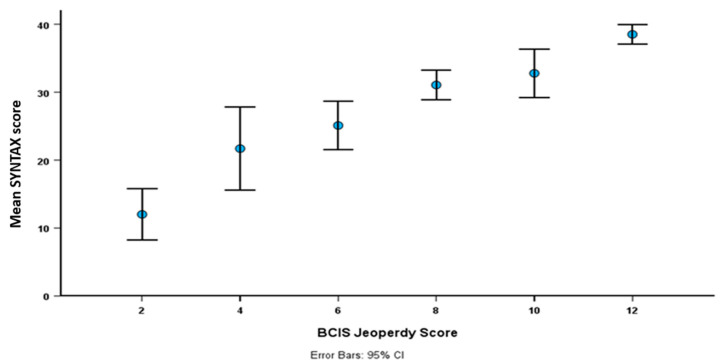
Relationship between SYNTAX and BCIS-JS in the whole cohort.

In the whole cohort, there was a modest relationship between BCIS-JS and SYNTAX score (Spearman r = 0.44, *p* < 0.001) and this relationship was even weaker in patients with diabetes (Spearman r = 0.32, *p* < 0.001). Notably, the variation in SYNTAX scores became wider in patients with low compared to high BCIS-JS scores (Figure 1).

**Figure 2:**
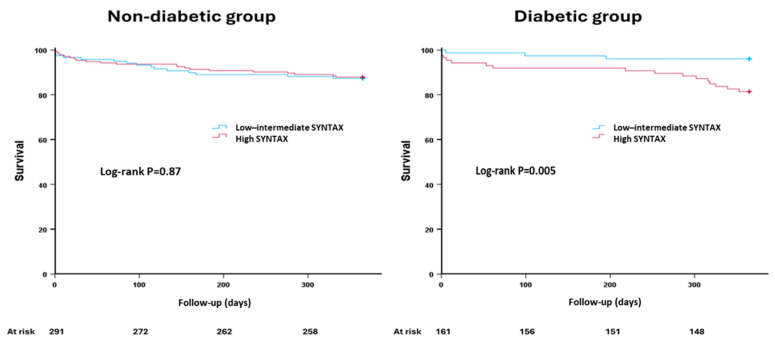
The primary endpoint in patients undergoing complex percutaneous coronary intervention according to diabetes status. Kaplan–Meier curves comparing cumulative incidence of all-cause mortality in patients with high versus low–intermediate SYNTAX scores.

**Figure 3:**
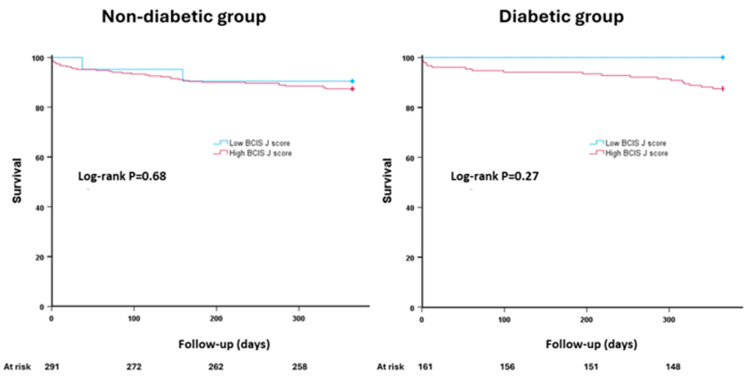
The primary endpoint in patients undergoing complex percutaneous coronary intervention according to diabetes status. Kaplan–Meier curves comparing cumulative incidence of all-cause mortality in patients with high versus low BCIS-JS scores.

The primary endpoint was reported in 55 patients (12.2%) in the whole cohort. There was no difference between diabetic and non-diabetic patients in procedural, in-hospital, one month mortality, or one-year mortality (Table 2). The primary endpoint was comparable in the non-diabetic group irrespective of the score used to define complex CAD. Non-diabetic patients with high SYNTAX scores had similar mortality rates at 12 months compared to those with low SYNTAX scores [12.1% vs. 12.7%, hazard ration (HR) 0.95, 95% confidence interval (CI) (0.49–1.84), *p* = 0.87] (Figure 2). Likewise, non-diabetic patients with high versus low BCIS-JS scores had similar incidence of the primary endpoint [12.6% vs. 9.5%, HR 1.35 95% CI (0.32–5.61), *p* = 0.68] (Figure 3).

On the other hand, there was a differential prognostic outcome in the diabetic group. The primary endpoint was more frequently reported in diabetic patients with high versus low SYNTAX scores [18.6% vs. 4.0%, HR 4.96, 95% CI (1.44–17.03), *p* = 0.011] (Figure 2). This observation was not evident in diabetic patients with high versus low BCIS-JS scores [12.5% vs. 0%, HR 22.04, 95% CI (0.01–101.41, *p* = 0.47] (Figure 3).

## 4. Discussion

The main findings of our study can be summarised as follows: (1) there was no difference in procedural, in-hospital, or one year mortality between diabetic and non-diabetic patients; (2) there were differential prognostic outcomes using SYNTAX score according to diabetic status; (3) a high BCIS-JS score did not differentiate patients at increased risk of mortality in either non-diabetic or diabetic patients.

Diabetes remains a major challenge for patients with complex CAD undergoing revascularization, particularly with PCI [9,21]. Poor glycaemic control was associated with higher stent failure and risk of restenosis in diabetic patients [15,21]. Additionally, the quality of life in patients who underwent PCI remained sub-optimal compared to patients who underwent CABG [22]. Interestingly, our study did not highlight worse clinical outcomes in diabetic versus non-diabetic patients. Importantly, there was no difference in the complexity of CAD between the two groups using either SYNTAX or BCIS-JS scores. It is of note that our cohort included high-risk patients who had been turned down for surgery with a potentially competitive risk of mortality in both diabetic and non-diabetic patients. Moreover, the diabetic patients were younger, with a lower percentage of chronic obstructive pulmonary disease and previous stroke. Such a relatively favourable profile may have offset the increased risk associated with diabetes and high body mass index. Collectively, this may explain the lack of mortality difference between the two groups.

The relationship between BCIS-JS and SYNTAX was only modest. There are anatomical factors that are included in the calculation of SYNTAX score that are not included in the BCIS-JS. Features such as lesion length, tortuosity, and calcifications are recognised to add procedural complexity but are not included in the BCIS-JS. Additionally, the presence of chronic total occlusion (CTO) has a differential impact on the two scoring systems, and it significantly increases the SYNTAX scores more than the BCIS-JS scores. The degree of stenosis for any lesion that is required to be included is also different between the two scoring systems [3].

The primary endpoint, defined as mortality at 12 months, was only significantly different in patients with diabetes using the SYNTAX score. Unlike BCIS-JS, diabetic patients with high SYNTAX scores had a five-fold greater likelihood of death at 12 months compared to those with low SYNTAX scores. This observation was not evident when using BCIS-JS to define complex CAD in diabetic patients. Whilst type 1 error remains a possibility given the relatively low number of diabetic patients and low BCIS-JS, potential biological reasons may explain our findings. The sample size of the subgroup of diabetic patients with low BCIS-JS was underpowered, precluding any definitive conclusion from the analysis. Nonetheless, diabetic patients tend to have large plaque burden and more diffuse, tortuous, and calcified CAD [23]. The prevalence of CTO is higher in diabetic patients and is associated with worse clinical outcomes in diabetic versus non-diabetic patients [24,25,26]. These features of atherosclerotic disease in diabetes will inevitably contribute to long-term outcomes but more importantly are captured within the SYNTAX scoring. Additionally, such plaque characteristics will add more procedural complexity related to lesion preparation, stent delivery, and optimisation. Collectively, this will result in sub-optimal stent results and a high likelihood of stent failure and major adverse cardiovascular events. In contrast, the BCIS-JS predominately encompasses lesion locations without any granularity of other features of anatomical complexity. This would explain the difference in the proportion of patients who were considered to have high SYNTAX versus high BCIS-JS scores. The presence of CTO, for example, would add more points to the complexity of SYNTAX score compared to non-CTO lesions. On the other hand, both CTO and non-CTO lesions would account for similar complexity using BCIS-JS. Furthermore, previous studies have defined high BCIS-JS using a relatively low threshold and, therefore, it is inevitable that the same patient may have high BCIS-JS but a low SYNTAX score. Our findings may have important clinical implications for risk stratification and treatment planning in patients with diabetes undergoing PCI for complex CAD. While the BCIS-JS has shown prognostic value in general, for PCI populations, including those with previous CABG, our results suggest that it may not be as effective in diabetic patients with high-risk features. Clinicians should exercise caution when using the BCIS-JS alone for risk assessment in this specific patient group.

Both BCIS-JS and SYNTAX did not provide prognostic insights in the non-diabetic group. The primary endpoint was comparable between the four subgroups (low versus high score of SYNTAX and BCIS-JS). This may be related to the pre-defined primary endpoint of all-cause mortality. The difference in clinical outcomes must be of a large magnitude to reflect a difference in mortality. Therefore, the complexity of CAD in non-diabetic patients does not appear to be a decisive factor in determining death at 12 months. The mortality rate was between 9 and 12% across the four sub-groups and the role of BCIS-JS and SYNTAX was relatively limited in non-diabetic patients. It is important to acknowledge that the outcomes observed in this study may be influenced by individual patient variability, particularly genetic factors that were not evaluated within the scope of our research. The response to treatment and overall prognosis could potentially differ among patients due to genetic predispositions or polymorphisms that affect cardiovascular health and treatment efficacy [27].

Our study has several limitations that need to be highlighted. This was a retrospective single-centre analysis, and the results need to be interpreted within the inherent limitation of the study’s design. Our study did not collect data on medical treatments, including antiplatelet, lipid-lowering drugs, and diabetic treatment. The primary endpoint was all-cause, rather than cardiovascular mortality. Similarly, our study did not include the risk of myocardial infarction and unplanned revascularization. This did not allow a deep-dive analysis of the role of BCIS-JS and SYNTAX scoring in both diabetic and non-diabetic patients. Finally, the proportion of patients with CTO was not presented in our study. This would have provided better understanding of the difference between the two scoring systems.

## 5. Conclusions

The present study demonstrates a modest correlation between SYNTAX and BCIS-JS systems, particularly among diabetic patients. Notably, the SYNTAX score, unlike the BCIS-JS, identified diabetic patients at an increased risk of 12-month mortality, underscoring its potential prognostic utility in this cohort. However, neither scoring system effectively differentiated mortality risk among non-diabetic patients. Further research is warranted to validate these findings, explore potential refinements to the scoring systems, and investigate their impact on long-term outcomes and additional endpoints beyond mortality.

## Figures and Tables

**Table 1:** Baseline clinical and procedural characteristics according to diabetes status.

	Whole Cohort (n = 452)	Non-Diabetic (n = 291)	Diabetic (n = 161)	*p* Value
Age	72 ± 11	73 ± 11	70 ± 11	0.001
Female gender	132 (29%)	79 (27%)	53 (33%)	0.20
BMI	29 ± 6	27 ± 5	31 ± 7	<0.001
Stable angina	206 (46%)	137 (47%)	69 (43%)	0.39
Previous MI	115 (25%)	70 (24%)	45 (28%)	0.36
PVD	77 (17%)	44 (15%)	33 (21%)	0.14
Previous CVA	47 (11%)	38 (13%)	9 (6%)	0.013
Previous CABG	35 (8%)	21 (7%)	14 (9%)	0.56
COPD	76 (17%)	58 (20%)	18 (11%)	0.043
Preserved LV	205 (52%)	136 (53%)	69 (51%)	0.65
CKD	159 (36%)	91 (32%)	68 (43%)	0.022
Euro Score II	6 ± 6	6 ± 6	7 ± 7	0.24
Left main	190 (42%)	127 (44%)	63 (39%)	0.35
Number of lesions	2 (1–3)	2 (1–3)	2 (1–3)	0.85
Number of stents	3 (2–4)	2 (1–3)	2 (1–3)	0.93
SYNTAX score	33 ± 13	34 ± 13	33 ± 12	0.41
SYNTAX II score	30 ± 17	31 ± 16	28 ± 17	0.08
High SYNTAX	259 (57%)	173 (60%)	86 (53%)	0.21
BCIS-JS	12 (8–12)	12 (8–12)	12 (8–12)	0.99
High BCIS-JS	422 (93%)	270 (93%)	152 (94%)	0.51
Optimal medical treatment	305 (67%)	185 (64%)	120 (75%)	0.12

BMI—body mass index; CABG—coronary artery bypass graft; CKD—chronic kidney disease; COPD—chronic obstructive pulmonary disease; LV—left ventricle; MI—myocardial infarction.

**Table 2:** Clinical outcomes according to diabetes status.

	Whole Cohort (n = 452)	Non-Diabetic (n = 291)	Diabetic (n = 161)	*p* Value
Procedural complications	29 (6.4%)	18 (6.2%)	11 (6.8%)	0.79
In-hospital death	10 (2.2%)	7 (2.4%)	3 (1.9%)	0.71
Death at one month	19 (4.2%)	6 (3.7%)	13 (4.5%)	0.71
One year mortality	55 (12.2%)	36 (12.4%)	19 (11.8%)	0.86

## Data Availability

The original contributions presented in this study are included in the article. Further inquiries can be directed to the corresponding author.
